# Pause the Draws: A Quality Improvement Project to Minimize Unnecessary Liver Function Tests in a Small District General Hospital

**DOI:** 10.7759/cureus.97681

**Published:** 2025-11-24

**Authors:** Priyanka Kannan, Sanjana Mathew, Bethan Griffith

**Affiliations:** 1 Medicine, Leighton Hospital, Mid Cheshire Hospitals NHS Foundation Trust, Crewe, GBR; 2 Medicine, Arrowe Park Hospital, Wirral University Teaching Hospital NHS Foundation Trust, Liverpool, GBR; 3 Acute Medicine, Leighton Hospital, Mid Cheshire Hospitals NHS Foundation Trust, Crewe, GBR

**Keywords:** carbon footprint, cost savings, environment, liver function test, quality improvement, sustainable healthcare, unnecessary blood tests

## Abstract

Introduction

Blood tests are essential for diagnosis and monitoring in hospital settings, yet many are performed without clear clinical benefit. Unnecessary blood testing can negatively affect patients by prolonging hospital stays, disrupting sleep, and prompting further investigations that may not be needed, while also adding avoidable financial costs and carbon emissions. This quality improvement project focused on reducing inappropriate liver function tests (LFTs) in two medical wards, following the Royal College of Pathology (RCPath) guidelines, with the aim of halving the proportion of inappropriate tests. Our initial audit identified that 28% of LFTs performed on the wards were unnecessary, demonstrating a clear opportunity for improvement.

Methods

The extent and causes of unnecessary LFTs were identified across two medical wards. Following the RCPath guidelines, any LFT repeated within 72 hours of a previous test was considered inappropriate unless an exclusion criterion applied. Interventions were implemented over four Plan-Do-Study-Act (PDSA) cycles. Weekly data were collected on the total and inappropriate number of LFTs for every patient present on the wards on Fridays. For each inappropriate LFT, the request information was reviewed to assess the rationale and inform subsequent PDSA cycles.

Results

Across the four PDSA cycles, inappropriate LFTs decreased from 72 out of 257 (28.0%) to 18 out of 98 (18.4%). The weekly LFT carbon footprint decreased from 263.34 kgCO₂e to 83.2 kgCO₂e. The weekly cost of unnecessary LFTs was reduced from £140.79 to £44.46.

Conclusions

The reduction of inappropriate LFTs yielded measurable clinical, environmental, and financial benefits. Patients avoided unnecessary blood tests that could complicate or prolong their admissions. Cost savings were demonstrated, and the hospital’s carbon footprint was reduced. The project’s impact, achieved through simple and practical interventions, highlights that physician education is central to optimizing blood testing.

## Introduction

Blood tests are an integral part of hospital care, supporting clinicians in diagnosis, monitoring disease severity, and assessing treatment response [1]. However, these benefits must be weighed against potential drawbacks, including patient discomfort, misinterpretation of results that may affect management, and the financial, labor, and environmental costs associated with unnecessary phlebotomy. Studies have reported that 40-60% of blood tests performed may be unnecessary [2].

Unnecessary blood tests can prolong hospital stays, disrupt patient sleep, and trigger further unneeded investigations. They also increase costs for healthcare institutions and contribute to higher greenhouse gas emissions [3].

This quality improvement project (QIP) aimed to reduce inappropriate liver function tests (LFTs), defined as those repeated within 72 hours without meeting the Royal College of Pathology (RCPath) exception criteria, by 50% (from 28% to 14%) over a five-week period across two medical wards. Secondary objectives included reducing total LFT requests, associated costs, and the carbon footprint from unnecessary testing.

The project was presented as a poster at “The Future of Healthcare” conference, organized by the North West Foundation Forum on June 14, 2025 in Manchester.

## Materials and methods

This QIP was conducted using a Plan-Do-Study-Act (PDSA) approach at Leighton Hospital, a small district general hospital in the North West Deanery, England, United Kingdom, between February and March 2025. The project was implemented across two general medicine inpatient wards: Ward 5, a gastroenterology ward, and Ward 14, a general medical ward. These wards were selected to include one specialist and one general medical ward to capture a representative sample of inpatient care.

**Figure 1:**
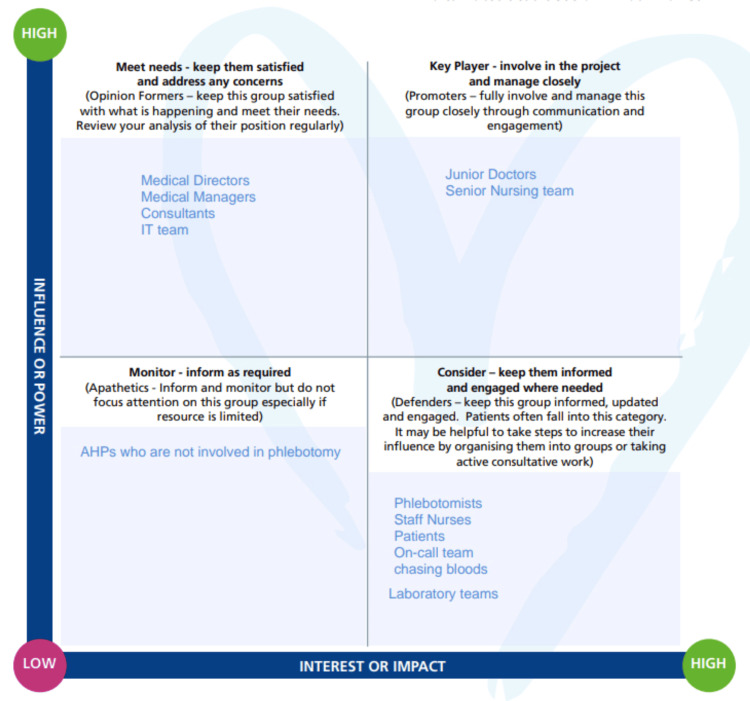
Stakeholder analysis highlighting populations most affected by the project and those with the greatest power to influence changes AHP, allied health professional

A stakeholder analysis was performed to identify individuals who could meaningfully contribute to improving current practices and to determine those most likely to be affected by the project. The results of this analysis are presented in Figure 1.

After identifying the stakeholders, a preliminary audit was conducted to quantify the total number of LFTs requested and the proportion considered inappropriate across the two medical wards during patients’ inpatient stays. It should be noted that Ward 5 included a mix of general medical patients, who were not excluded from the study. On a single day in January 2025, there were a total of 62 patients across both wards.

An inappropriate LFT was defined as one performed within 72 hours of a previous LFT in patients who did not meet the exception criteria advised by the RCPath. Four scenarios allow for repeating an LFT within 72 hours: patients on total parenteral nutrition (TPN), patients in critical care units, patients with acute liver injury, and patients presenting with acute poisoning from potentially hepatotoxic drugs [4].

The preliminary audit revealed that 257 LFTs were requested for 62 patients during their admissions. Of these, 152 were repeated within 72 hours, and 72 (28.01%) were classified as inappropriate. For each inappropriate LFT, the reason for the request was documented. Examples included “acute confusion,” “monitoring sepsis of unknown source,” “AKI monitoring,” “treated for pyelonephritis,” “monitoring and screening as per Dr. X,” and simply “repeat.”

**Figure 2:**
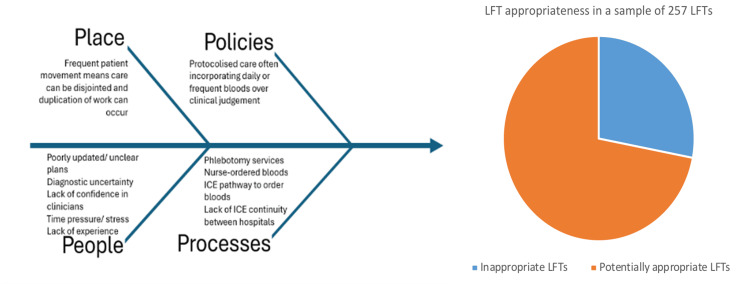
Fishbone diagram illustrating factors contributing to inappropriate LFT requests, accompanied by a pie chart showing LFT appropriateness in a random sample of 257 requests ICE, integrated clinical environment; LFT, liver function test

Following discussions with physicians from both wards, several potential triggers for inappropriate LFTs were identified, as summarized in Figure 2.

**Figure 3:**
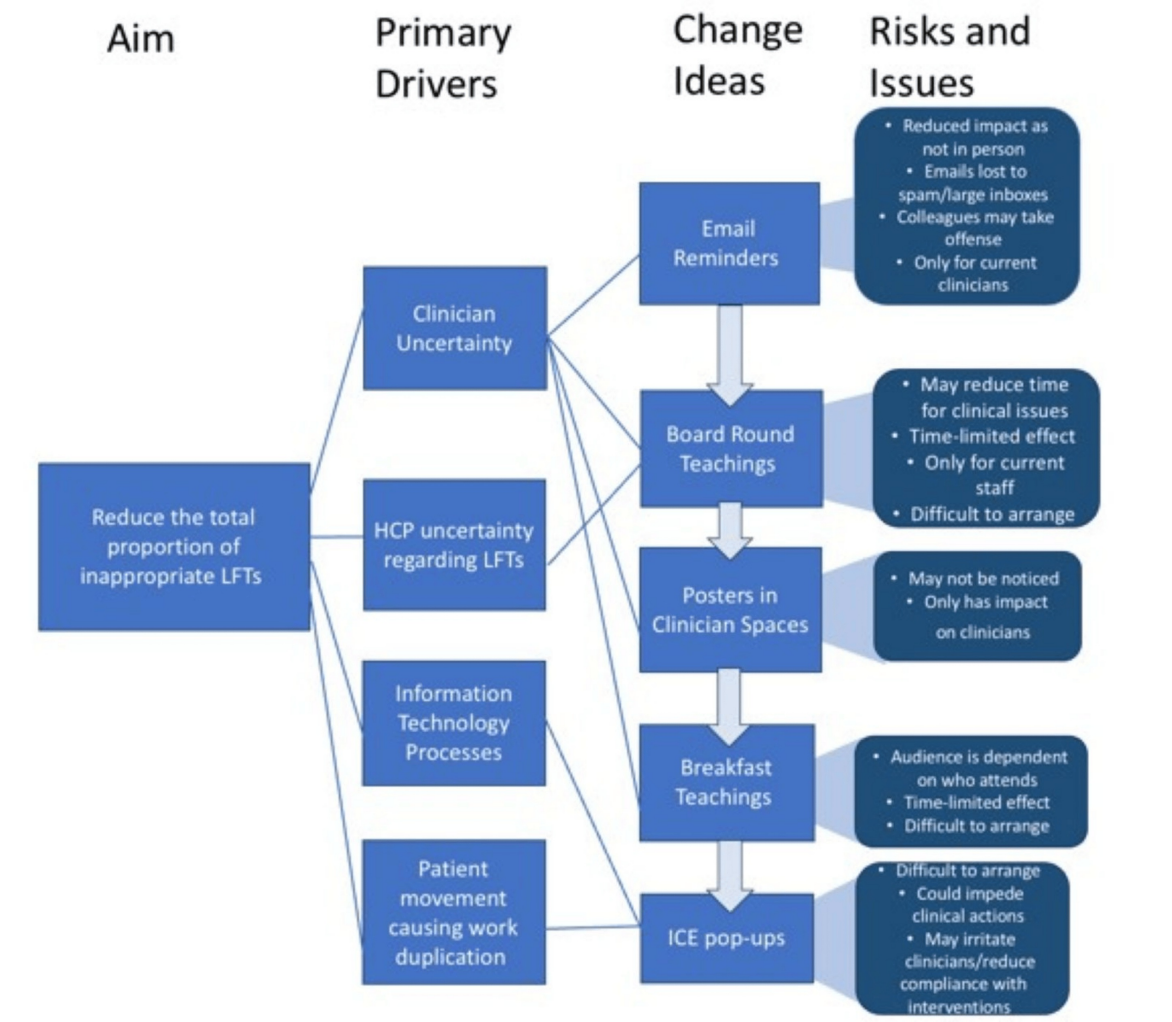
Driver diagram illustrating primary drivers contributing to the high number of LFTs and potential interventions targeting these drivers HCP, health care professional; ICE, integrated clinical environment; LFT, liver function test

A specific, measurable, achievable, relevant, and time-bound (SMART) aim was established to minimize inappropriate LFTs. The primary goal was to reduce the total proportion of inappropriate LFTs on Wards 5 and 14 from 28% to 14% within a five-week period. A driver diagram, shown in Figure 3, was developed to identify the primary drivers and potential change ideas that could address them. Risks and issues associated with these targeted interventions were also highlighted. In this diagram, ICE refers to the software used by the trust to request blood tests.

Secondary aims of the project included reducing the total number of LFTs ordered, decreasing the carbon footprint generated by processing inappropriate LFTs, and lowering associated financial expenditures.

Data were collected weekly on the total and inappropriate number of LFTs for every patient present on the wards on the Friday of each week. For patients who remained on the same ward in the following week, LFTs captured in the previous week were excluded, as those tests would not have been affected by any interventions. Consequently, the sample size varied across intervention cycles, and the percentage reduction in inappropriate LFTs was calculated to assess improvement. For each inappropriate LFT, the request information was reviewed to determine the reasons and guide subsequent PDSA cycles.

The first intervention involved email reminders to resident doctors and discussions during morning and afternoon board rounds, where doctors and nurses reviewed patient plans, including outstanding blood tests. A five-day period was allowed before data collection; interventions were implemented on Monday of the week (Week 0), and data were collected on Friday.

The second round of interventions included repeat emails to resident doctors, reflecting newly rotated staff in the department. Additionally, Post-it notes summarizing guidelines for repeating LFTs within 72 hours were placed on doctors’ desks in both wards. This intervention was also applied on Monday of Week 1, with data collected on Friday of the same week. At this stage, it was noted that weekly interventions appeared too brief to produce measurable change.

**Figure 4:**
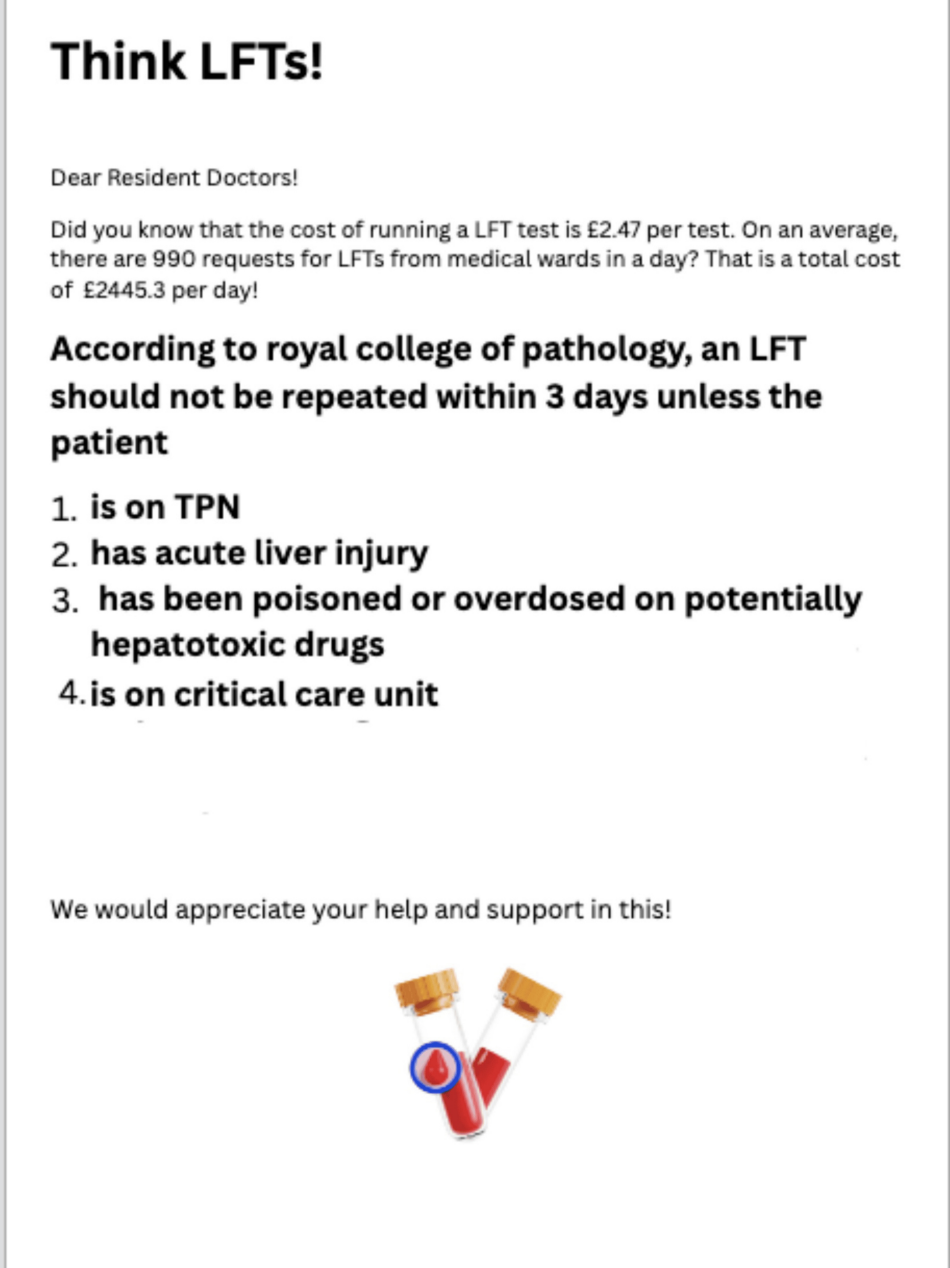
Poster implemented during the third cycle of intervention LFT, liver function test; TPN, total parenteral nutrition

For the third cycle, the data collection interval was extended to two weeks; the intervention was applied on Monday of Week 2, and data were collected on Friday of Week 3. Additionally, a quick, informative poster was displayed on poster boards, as shown in Figure 4.

The fourth cycle reinforced all previous interventions, including informal discussions with colleagues and re-sending reminder emails. This was implemented on Monday of Week 4, and data were collected on Friday of Week 5.

Data collection was performed by two team members on the Friday of each intervention week. For example, one member collected data from the gastroenterology ward after Cycle 1, while the other collected data from the general medical ward. Patient lists for each ward were printed, and for each patient, the date of the current admission was noted in the electronic medical record. LFTs were identified using the blood test filter in the online system. Tests requested from the critical care unit were excluded, in accordance with the exclusion criteria.

An Excel spreadsheet (Microsoft Corporation, Redmond, WA, USA) was created with the following columns: Ward, Hospital number, Total number of LFTs requested, Repeated LFTs within 72 hours, Number of inappropriate LFTs, and Reason provided for inappropriate LFT requests. The dates of requests were recorded, and repeated LFTs within 72 hours were manually counted while simultaneously populating the spreadsheet.

For each repeated LFT not from the critical care unit and lacking an appropriate reason (e.g., TPN monitoring), the team reviewed the patient’s paper notes from that morning’s ward round. The qualified clinicians on the team then determined whether the request met any of the remaining three exception criteria. LFTs that did not meet these criteria were classified as inappropriate. Inter-rater reliability was ensured by cross-checking each ward’s data, and a count of inappropriate LFTs was documented only if both team members agreed on its classification. The two members also alternated wards during data collection to minimize bias.

The cost of LFTs was calculated by multiplying the total or inappropriate number of tests by £2.47, representing the cost per test panel (total bilirubin, albumin, alkaline phosphatase (ALP), and alanine transferase (ALT)). This cost information was provided by the lead biochemical scientist at Leighton and Macclesfield Hospitals, whose contributions are acknowledged.

The estimated carbon footprint was calculated by multiplying the number of inappropriate LFTs by 462 g CO₂e, representing the emissions associated with processing a liver panel, which includes the international normalized ratio and partial thromboplastin time in addition to the liver panel analytes [3]. Although no literature was available to calculate carbon emissions for the liver panel alone, this estimate was deemed important to understand the environmental impact of unnecessary LFTs.

## Results

Baseline data showed that a total of 257 LFTs were requested across two medical wards during the inpatient stays of 62 patients. Of these, 72 LFTs (28.01%) were deemed inappropriate according to RCPath guidelines. Following the four intervention cycles described in the Materials and Methods section, the proportion of inappropriate LFTs decreased to 18 (18.36%). The total number of LFTs was reduced from 257 to 98 by the final week of intervention, representing nearly a two-thirds reduction. Similarly, the number of inappropriate LFTs decreased from 72 to 18 by the end of the last cycle.

The impact of these inappropriate LFTs can be quantified. Each LFT, which includes albumin, bilirubin, ALP, and ALT, costs £2.47, as reported by the lead biochemical scientist at the trust. At baseline (Week 0), the trust spent a total of £634.79 on LFTs during these patients’ inpatient stays, of which £177.84 was attributed to inappropriate LFTs.

The environmental impact was also estimated. Each LFT processed is associated with a carbon footprint of 462 g CO₂e [3]. Therefore, the baseline LFTs produced approximately 118.7 kg CO₂e, of which around 33.3 kg CO₂e was attributable to inappropriate LFTs.

During the first intervention cycle, regular board round discussions were conducted, and informational emails were sent to all resident doctors on the wards. In that week, 43 inappropriate LFTs were requested out of 149 total LFTs (28.86%). Although the proportion of inappropriate LFTs slightly increased, there was a reduction in both the total number of LFTs and the number of inappropriate LFTs, indicating initial evidence of cost savings. These findings are summarized in Table 1.

**Table 1:** Raw figures of total LFTs, LFTs repeated within 72 hours, number of inappropriate LFTs, percentage of inappropriate LFTs, and cost wasted on inappropriate LFTs LFT, liver function test

Week	Total number of LFTs	Number of LFTs repeated within 72 hours	Number of inappropriate LFTs	Percentage of inappropriate LFTs	Money wasted on inappropriate LFTs
0 (initial audit)	257	157	72	28.01%	£135.85
1	149	78	43	28.86%	£106.21
2	129	68	33	25.58%	£81.51
3	73	27	18	24.66%	£44.46
4	98	37	18	18.36%	£44.46

During Week 2 of intervention, the team sent informational emails to newly rotated doctors, placed Post-it notes with guidelines on doctors’ desks, and continued informal discussions with colleagues. Following this intervention, inappropriate LFTs decreased to 33 out of 129 requests (25.58%).

In Week 3, posters were printed and displayed on bulletin boards in both wards, and electronic copies were emailed to resident doctors. The intervention cycle length was extended by one week to allow doctors time to adopt the RCPath guidelines. This resulted in a further reduction in inappropriate LFTs to 18 out of 73 new requests (24.66%). For consistency in graphical representation, the weeks were labeled consecutively.

The final week of data collection reinforced all previous interventions and allowed a two-week interval to observe changes. Inappropriate LFTs decreased to 18 out of 73 requests (18.36%). The cycles were concluded at this point, approaching the target of 14%.

**Figure 5:**
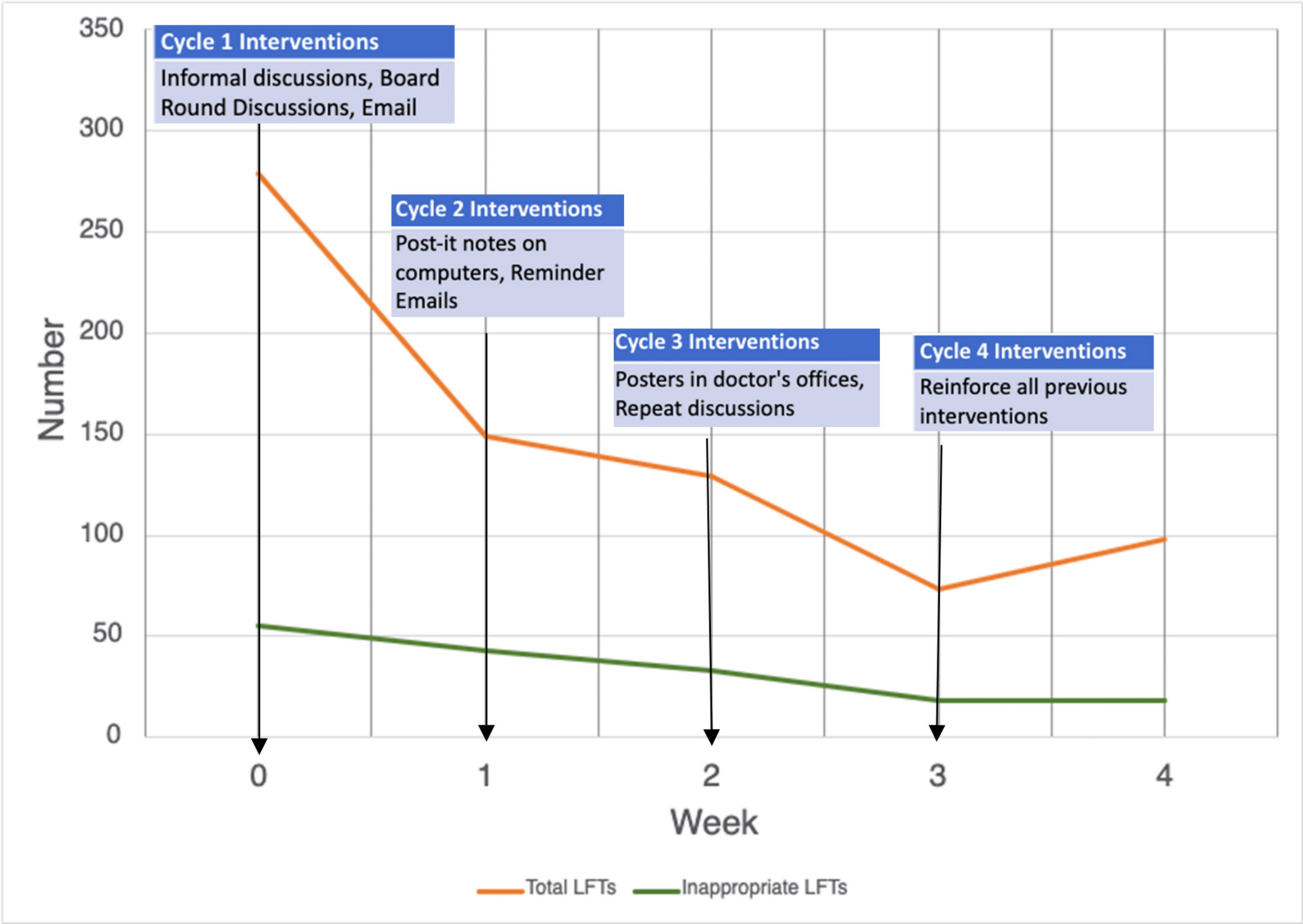
Number of LFTs is represented on the y-axis and weeks on the x-axis The orange line represents the total number of LFTs requested per week, and the green line represents the number of inappropriate LFTs requested weekly. LFT, liver function test

Figure 5 illustrates the downward trend in both total LFTs requested and inappropriate LFTs from Week 0 (pre-intervention) to Week 5, along with the corresponding intervention cycles.

**Figure 6:**
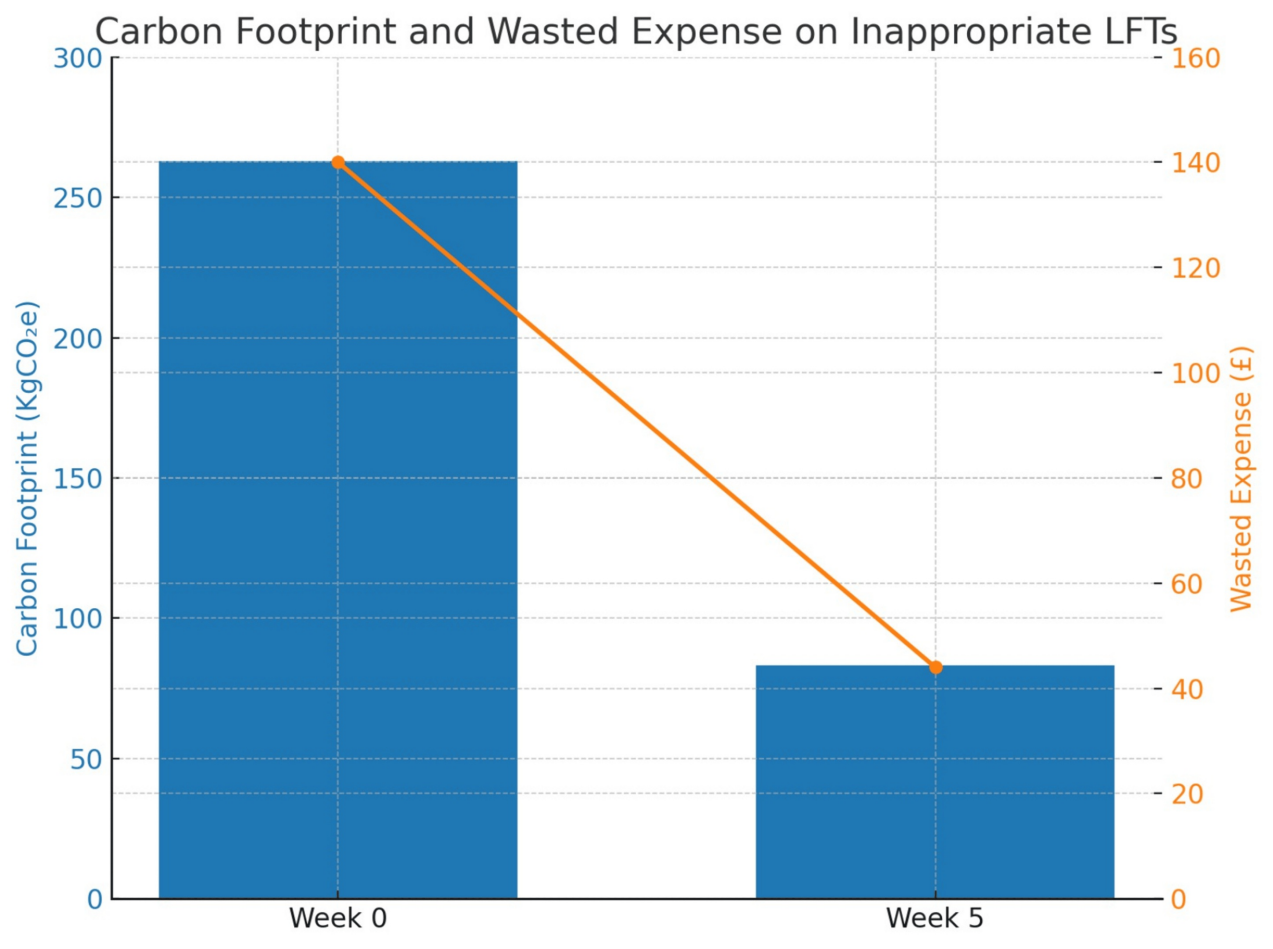
Carbon footprint and wasted expense on inappropriate LFTs The left y-axis represents the carbon footprint (kg CO₂e) from processing inappropriate LFTs, and the right y-axis represents the associated wasted expense (£). The orange line across the bar graphs illustrates the reduction in costs related to processing inappropriate LFTs. LFT, liver function test

Figure 6 illustrates two outcomes. The blue bars represent the reduction in carbon footprint from processing inappropriate LFTs, decreasing from 263.84 kg CO₂e to 83.2 kg CO₂e, which is equivalent to the carbon dioxide emitted from driving a car for approximately 800 miles [5]. The diagonal orange line across the graph indicates the reduction in weekly costs of inappropriate LFTs, which decreased from £140.79 to £44.46.

These results demonstrate that simple, low-cost interventions can significantly reduce environmental impact and generate financial savings for healthcare institutions. Although the target of reducing inappropriate LFTs to 14% was not fully achieved, this project highlights the practical application of QI methodology and shows that small, targeted interventions can lead to meaningful improvements.

## Discussion

Interpretation and implications

This study demonstrates a reduction in the proportion of inappropriate LFT requests, accompanied by corresponding decreases in both carbon footprint and financial expenditure following the PDSA cycles. The interventions led to an estimated cost savings of nearly £100 per week across the two wards. When extrapolated across multiple wards and on a daily basis, this represents a substantial potential financial saving. Resources spent on unnecessary LFTs could be redirected toward more clinically valuable investigations.

The reduction in carbon footprint from decreased inappropriate LFT use is equivalent to the CO₂ emissions generated by driving a car approximately 800 miles. Expanding these interventions to other medical wards could further enhance sustainability efforts and support the NHS Long Term Plan to achieve a Net Zero NHS. This plan includes targets for reducing the NHS carbon footprint by 80% between 2028 and 2032, with the ultimate goal of achieving net-zero emissions by 2045 [6].

Comparison with existing literature

A similar study conducted by the Pediatric ICU team at Great Ormond Street Hospital found that LFTs were often repeated daily even when previous results were normal. The intervention prevented unnecessary LFTs and resulted in cost savings of approximately £4,000-£5,000 per month [7].

In general practice, another study aimed to reduce the number of full liver panels (7 analytes) in favor of single ALT tests for patients on statins. It estimated that a 20% reduction in full liver panels could save approximately £8.4 million per year in England [8].

A five-year multicenter retrospective observational study in Australia showed that, despite clear guidelines advising against repeating LFTs within 48-72 hours, nearly half of repeat LFTs were ordered within a median interval of 25.6 hours. The study concluded that further interventions were needed to improve adherence to recommended minimum testing intervals [9].

Another QIP across four hematology centers in the UK used a PDSA approach to reduce inappropriate blood testing by introducing a structured testing schedule. They achieved a median reduction of 24.7% in inappropriate tests and estimated annual cost savings of up to £38,438 [10].

Together, these studies highlight the financial benefits of reducing inappropriate blood testing and reinforce the potential for simple, targeted interventions to improve efficiency and sustainability in clinical practice.

Limitations and strengths

Several factors may have influenced the study results. The varying length of inpatient stays could have affected opportunities for LFT repetition, as patients discharged within three days may not have had repeat tests. This variability is unpredictable because patient diagnoses differ across wards. For example, a gastroenterology ward may be more likely to order daily LFTs, even for general medicine patients without liver-related issues.

Another limitation is the unequal length of time for the final intervention cycle compared to earlier cycles. While this may have affected protocol standardization, it also demonstrated that longer intervention periods are needed to observe measurable change. Variations in weekly patient mix may have further influenced results. For instance, a week with a high number of paracetamol overdose admissions could disproportionately affect inappropriate LFT counts.

Frequent locum coverage may also have influenced results, as these doctors were unaware of the project, a common real-world implementation challenge. Additionally, the absence of a control group limits the ability to distinguish whether changes were due to the interventions or natural variation. Different wards have inherently different patient populations and lengths of stay; for example, geriatric wards often have long admissions, whereas acute medical wards may have rapid patient turnover. Consequently, statistical analyses, such as analysis of variance, could not be performed, and results likely demonstrate association rather than causation.

Despite these limitations, QIPs focus on sustainable improvements in patient care rather than experimental validation. Several strengths underpin this project. The structured use of PDSA cycles enabled targeted engagement with key stakeholders and interventions addressing the most significant sources of inappropriate testing. Early and continuous data collection allowed timely trend identification and iterative refinement of interventions. Cross-checking data by a second reviewer ensured internal validity. The project also demonstrated that sustained physician education through regular, informal discussions is highly effective in driving change. Interventions were simple, low-cost, and easy to implement, enhancing generalizability to other wards or hospitals.

Future recommendations 

To achieve meaningful and sustained change, electronic prompts within the hospital’s blood test ordering system could be implemented. For example, if an LFT is requested within 72 hours of a prior test, a pop-up could require clinicians to justify the request. This encourages reflection on test necessity and reduces unnecessary repetitions. Evidence supports this approach; a systematic review of 41 studies found that EMR-based interventions were most effective in reducing inappropriate blood test requests [11]. Similar measures have already been applied for thyroid function tests, iron studies, and coagulation screens in this hospital.

Additionally, the hospital’s continuous improvement team has logged this project, allowing future rotating doctors to continue it. The team provides awards ranging from bronze to gold, with gold recognizing sustained projects that have spread across wards. This system supports sustainability, provides incentives, and saves time by allowing new doctors to build on existing stakeholder analyses and fishbone diagrams rather than starting PDSA cycles from scratch.

## Conclusions

This QIP demonstrated that targeted interventions using a PDSA approach reduced the number of LFTs ordered, lowering both costs and carbon emissions. These findings highlight how diagnostic stewardship can be improved while supporting healthcare sustainability goals. The project’s impact, achieved through simple and practical interventions, emphasizes that physician education is central to optimizing blood testing. Although conducted over a short timeframe and limited to two medical wards, the approach can be extrapolated to other wards and reaudited in subsequent months to ensure sustained improvement. Continuous reauditing and integration of these changes into the electronic medical record would further enhance their effectiveness. This QIP can also be applied to other specialty wards across multiple sites by following the RCPath guidelines.
